# Integrative Transcriptomic and Small RNA Sequencing Reveals Immune-Related miRNA–mRNA Regulation Network for Soybean Meal-Induced Enteritis in Hybrid Grouper, *Epinephelus fuscoguttatus*♀ × *Epinephelus lanceolatus*♂

**DOI:** 10.3389/fimmu.2020.01502

**Published:** 2020-08-06

**Authors:** Yuanfa He, Guanlin Ye, Shuyan Chi, Beiping Tan, Xiaohui Dong, Qihui Yang, Hongyu Liu, Shuang Zhang

**Affiliations:** ^1^Laboratory of Aquatic Animal Nutrition and Feed, Fisheries College, Guangdong Ocean University, Zhanjiang, China; ^2^Aquatic Animals Precision Nutrition and High-Efficiency Feed Engineering Research Center of Guangdong Province, Zhanjiang, China; ^3^Key Laboratory of Aquatic, Livestock and Poultry Feed Science and Technology in South China, Ministry of Agriculture, Zhanjiang, China; ^4^Southern Marine Science and Engineering Guangdong Laboratory, Guangdong Ocean University, Zhanjiang, China

**Keywords:** hybrid grouper, inflammation, RNA-seq, intestinal health, immune response

## Abstract

A 10-week feeding experiment was conducted to reveal the immune mechanism for soybean meal-induced enteritis (SBMIE) in hybrid grouper, Epinephelus fuscoguttatus ♀ × Epinephelus lanceolatus ♂. Four isonitrogenous and isolipidic diets were formulated by replacing 0, 10, 30, and 50% fish meal protein with soybean meal (namely FM, SBM10, SBM30, and SBM50, respectively). The weight gain rate of the SBM50 group was significantly lower than those of the other groups. Plica height, muscular layer thickness, and goblet cells of the distal intestine in the SBM50 group were much lower than those in the FM group. The intestinal transcriptomic data, including the transcriptome and miRNAome, showed that a total of 6,390 differentially expressed genes (DEGs) and 92 DEmiRNAs were identified in the SBM50 and FM groups. DEmiRNAs (10 known and 1 novel miRNAs) and their DE target genes were involved in immune-related phagosome, natural killer cell-mediated cytotoxicity, Fc gamma R-mediated phagocytosis, and the intestinal immune network for IgA production pathways. Our study is the first to offer transcriptomic and small RNA profiling for SBMIE in hybrid grouper. Our findings offer important insights for the understanding of the RNA profile and further elucidation of the underlying molecular immune mechanism for SBMIE in carnivorous fish.

## Introduction

In recent years, the global contribution of fish meal to aquafeeds has sharply reduced (1). As a result, soybean meal, which is considered one of the most hopeful candidates for fish meal replacement, can partially or fully replace the fish meal but introduces many anti-nutritional factors. Consequently, fish enteritis induced by plant proteins has become one of the main challenges for sustainable aquaculture (2), and this occurs in a dose-dependent manner (1, 3).

Soybean meal-induced enteritis (SBMIE) has been found in many commercial fish species such as Atlantic salmon (*Salmo salar*) (4), grass carp (*Ctenopharyngodon idella*) (5), and turbot (*Scophthalmus maximus* L.) (6, 7), and mainly occurs in carnivorous fish (4, 6, 7). The symptoms of SBMIE are most apparent in the posterior/distal intestine of turbot (6, 7) and grass carp (5), the most important mucosal immune organ (8). Fish SBMIE has been found to be accompanied by a decrease in the height of villi and microvilli, downregulation of tight junction protein *claudin-4, occluding*, and *ZO-1* mRNA levels, and upregulation of pro-inflammatory cytokine genes, including *TNF-*α, *IL-1*β, *IL-8*, and *IL-16* (9–12), influencing the innate immune response. Zebrafish SBMIE is T cell-dependent and has a T helper (Th) 17 cytokine profile (13). What are the underlying immune mechanisms in fish SBMIE? The new omics technologies, including genomics, proteomics, and transcriptomics, have great potential for investigating and explaining the complex relationship between fish nutrition and immunity, both in intestine health and disease (14). At the genomic level, most components associated with T lymphocyte function have been identified in fish, suggesting that gut-associated lymphoid tissue has similar functionalities between fish and mammalian T lymphocytes (15). At the transcriptomic level, immune-related pathways of fish SBMIE have been gradually reported, showing that cytokine–cytokine receptor interaction, NOD-like receptor interaction, the intestinal immune network for IgA production, and the NF-kB, Jak-STAT, T-cell receptor, and TNF signaling pathways played key roles in response to SBM stress (3, 16). Fish meal replacement in fish diet by alternative protein sources could change the fish intestine proteome, including innate immune proteins (17).

The especially interesting fact is that miRNAs are involved in regulating intestine function, including epithelial cell growth (18), mucosal barrier function (19), and the development of gastroenteric diseases (20–22). An important aspect is that miRNA can also regulate mRNA expression in fish at a transcriptional level. Recently, studies of miRNAome in turbot intestinal function have reported that miRNAs contributed to the intestinal immune responses, preventing host infection, which the potential target genes of differentially expressed miRNAs were involved in multiple functional categories, including the RIG-I signaling pathway, immune defense/evasion, the toll-like receptor signaling pathway, and inflammatory responses (23). Also, it was found via small RNA sequencing that fish diet could affect the expression of intestinal miRNAs and target genes and immune-related pathways, including cell adhesion molecules, ECM–receptor interaction, the apoptosis signaling pathway, cytokine-cytokine receptor, and the VEGF signaling pathway (3, 24). However, there has been a lack of investigation of the underlying immune response by combining both transcriptomes and small RNA sequencing, and this requires further elucidation, especially in carnivorous fish.

Hybrid grouper (*Epinephelus fuscoguttatus*♀ × *Epinephelus lanceolatus*♂) is a carnivorous fish species that is the main farmed species in China due to its outstanding delicious taste and better growth rate and survival compared with the broodfish. Information on how nutrition influences the intestine health of hybrid grouper has been gradually accumulated in recent years (25–30). Our previous research used metabolomics technology to identify 17 potential markers of SBMIE in hybrid grouper (31). However, this study aims to reveal the immune-related miRNA-mRNA regulation network for SBMIE in hybrid grouper by integrative transcriptome and small RNA profiling from the perspective of molecular immunology and to provide another important insight to further solve the problem of fish intestinal health.

## Materials and Methods

### Experimental Diets

The use of hybrid grouper juveniles was approved by the Animal Research and Ethics Committees of Guangdong Ocean University, China. Four isonitrogenous and isolipidic diets were formulated to contain 0, 7.41, 22.24, and 37.07% of soybean meal (SBM) by replacing 0% (FM, control), 10% (SBM10), 30% (SBM30), and 50% (SBM50) of fish meal (FM) protein, respectively. The formulation of the basic experimental feeds is presented in Table 1. All ingredients were systematically mixed with lipid sources such as fish oil, soybean oil, and soybean lecithin and then purified water was added to produce a homogenous mixture. The dough was pelleted through a double helix extrusion mechanism (F-75, South China University of Technology, China). Feeds (2.5 mm diameter) were air-dried and then stored at −20°C until feeding.

**Table 1:** Ingredients and nutrient content of the diets (dry weight %).

**Ingredient**	**Experimental diet**			
	**FM**	**SBM10**	**SBM30**	**SBM50**
Fish meal	50.00	45.00	35.00	25.00
Peeled soybean meal	0.00	7.41	22.24	37.07
Vital wheat gluten	5.00	5.00	5.00	5.00
Wheat flour	14.60	14.60	14.60	14.60
Fish oil	4.11	4.52	5.33	6.14
Microcrystalline cellulose	14.33	11.49	5.80	0.10
Vitamin premix^a^	0.30	0.30	0.30	0.30
Mineral premix^b^	0.50	0.50	0.50	0.50
Lysine	0.00	0.06	0.18	0.30
Methionine	0.25	0.28	0.34	0.41
Arginine	0.33	0.26	0.13	0.00
Others^c^	10.68	10.68	10.68	10.68
Total	100.00	100.00	100.00	100.00
Nutrient levels (%)				
Crude Lipid	12.05	12.15	11.99	12.05
Crude protein	47.57	47.59	47.64	47.70
Lysine	2.75	2.85	2.75	2.76
Methionine	1.18	1.22	1.08	1.15
Arginine	2.28	2.28	2.34	2.46

a, b *The vitamin premix and mineral premix were obtained from Qingdao Master Biotech (Qingdao, China)*.

c *Others: casein, 4.00%; gelatin, 1.00%; soybean oil, 1.50%; soybean lecithin, 1.50%; vitamin C (35%), 0.05%; ethoxyquin, 0.03%; choline chloride, 0.50%; Ca (H_2_PO_4_)_2_, 2.00%*.

### Feeding Trial

Hybrid grouper Juveniles were obtained from a native species farm (Zhanjiang, China). All fish were adapted under the feeding system for 2 weeks by feeding with a commercial diet. Uniformly sized fish (mean initial weight ± SE = 17.01 ± 0.04 g) were randomly divided into four groups in triplicate, with 30 individuals in each fiberglass tank (300 L). The fish were slowly fed twice a day at 08:00 and 17:00 for 10 weeks. During the experiment, the water temperature fluctuated from 28 to 30°C, the dissolved oxygen concentration was kept at >7 mg/L, and ammonia and nitrate were kept at <0.03 mg/L.

### Sample Collection

Before the termination of the 10-week feeding experiment, the fish per tank were fasted for 24 h before collecting samples and were then counted and weighed to determine growth indexes, including weight gain rate, feed conversion ratio, and survival rate. After weighing, distal intestines of two fish per tank were collected and instantly transferred to 4% paraformaldehyde solution for histological examination. At the same time, distal intestines of another three fish per tank were collected as a single sample and instantly frozen in liquid nitrogen, then stored at −80°C for RNA extraction. Based on growth performance (see section Growth Performance) and histological examination, small RNA and transcriptome analyses were performed on distal intestine samples of the FM and SBM50 groups to ensure the maximum difference between samples. Thereby, the probability of detecting differential expression was increased between samples.

### Intestinal Morphology

The fixed distal intestine samples from FM and SBM50 groups were dehydrated in a series of graded ethanol and embedded in paraffin. Distal intestine sections (7 μm thick) from each sample were cut and then stained with hematoxylin/eosin. The sections were observed under an inverted microscope (Nikon, Japan), and 10 plicas and muscle layer thicknesses (MLT) were randomly selected per slice. Plica height (PH), plica width (PW), MLT, and the number of goblet cells (GC) per slice were measured using the image acquisition software (NIS Elements, version 4.60, Nikon, Japan).

### Transcriptome Sequencing and *de novo* Assembly

One microgram total RNA from the FM and SBM50 treatment groups was used for transcriptome library preparation. Total RNA was purified by beads containing oligo (dT). First-strand cDNA was then generated in a First-Strand Reaction System by PCR, and the second-strand cDNA was generated as well. The cDNA fragments with adapters were amplified by PCR, and the products were purified using AMPure XP Beads. The library was validated on the Agilent Technologies 2100 bioanalyzer for quality control. Transcriptome sequencing was carried out on a BGISEQ-500 platform (BGI-Shenzhen, China). Trinity (32) was used to achieve *de novo* assembly with clean reads, and Tgicl (33) was then used to cluster transcripts to Unigenes. The expected number of fragments per kilobase of transcript sequence per million base pairs sequenced (FPKM) was used to calculate the mRNA gene expression. Differentially expressed genes (DEGs) of two groups (SBM50 and FM) were quantified using the cutoff |log2FC| >1, *P* < 0.001 by DESeq R package (34, 35). After assembly, All-Unigenes were searched and annotated against the publicly available protein databases, including Nr (NCBI non-redundant protein sequences), Nt (NCBI non-redundant nucleotide sequences), KOG (EuKaryotic Orthologous Groups), Swiss-Prot, and GO (Gene Ontology). The pathway assignments were performed by sequence searches against the KEGG (Kyoto Encyclopedia of Genes and Genomes) database. KEGG terms with corrected *P*-values (*Q*-values) ≤ 0.05 were considered significant. Transcriptome (*de novo* assembly) sequencing data were deposited into the NCBI SRA database with the accession number SUB7020170.

### Construction and Sequencing of Small RNA Libraries

Six small RNA libraries were constructed from the FM and SBM50 treatment groups. Total RNA extraction was performed from the distal intestine using Trizol Reagent (Invitrogen, USA). Subsequently, 1 μg of total RNA per sample was used for small RNA sequencing. The quality of RNA samples was evaluated using the Agilent 2100 Bioanalyzer. Small RNA fractions were ligated to 3′ and 5′ adapter. Quantitative reverse transcription PCR (RT-PCR) was carried out on the adaptor-ligated small RNAs. PCR products were purified by QIAquick Gel Extraction Kit (Qiagen, Germany) and used for sequencing on the BGISEQ-500 platform (BGI-Shenzhen, China). Clean reads were obtained by cleaning low-quality tags, removing adapter sequences, and filtering adaptor-ligated contaminants and sequences fewer than 18 nucleotides (nt). The final reads were mapped to the *Hypoplectrus puella* (GCA_900610375.1) reference genome by Bowtie2 (36). Clean reads were compared against small RNAs (rRNA, scRNA, snoRNA, snRNA, tRNA, and mRNA) using the Rfam database to annotate small RNA sequences. Finally, the miRBase20.0 was used to look for know miRNA. The hairpin structures were used to predict novel miRNAs using miRDeep2 software (37).

miRNA expression levels were compared between the SBM50 and FM groups to identify differentially expressed miRNAs (DEmiRNAs). Firstly, data were normalized to obtain transcripts per million (TPM) values using the following formula: normalized expression = actual miRNA count/(total reads) ×1,000,000 (38). Fold-change values were then calculated based on log_2_ (SBM50/FM) expression. The corrected *P*-value corresponds to the differential gene expression test using the Bonferroni method (39). Differential miRNA expression between two groups was analyzed with DESeq software based on the following thresholds: *P*-value ≤ 0.01 and |log_2_ ratio| ≥ 1. RNAhybrid (40) and miRanda (41) software predicted the potential target genes of miRNA candidates, as described previously elsewhere (42, 43). The DAVID gene annotation tool was used for the KEGG pathway annotation of predicted miRNA targets. Small RNA sequencing data were deposited into the NCBI SRA database with the accession number SUB7175134.

### Network Analysis of DEmiRNA and DEG Interaction

Pearson's correlation coefficients between DEmiRNAs and their target genes were calculated using the correlation function in RStudio. To obtain the positive and negative correlations between two groups, the potential target genes of miRNAs were overlapped with the identified upregulated or downregulated DEGs, respectively. All of the relationship pairs between DEmiRNAs and their DE target genes were used to construct the interaction network using Cytoscape v.3.7.2 software.

### Validation by Real-Time Quantitative PCR (RT-qPCR)

RT-qPCR validation was carried out on the same samples used for transcriptome sequencing (*n* = 3). Primers were designed from the candidate gene sequences by premier 5.0 software and the online Primer-BLAST program. Primers used in this study are provided in Table S1. One microgram total RNA for RNA sequencing was reverse transcribed into cDNA. Real-time PCR assays were conducted on a CFX96 real-time PCR Detection System (Bio-Rad, Hercules, CA) with 5 μL SYBR Green Master Mix (Takara, China). β-actin was selected as the reference gene according to a previous study (26). The small RNA of the same samples used for sequencing was extracted using an RNAiso for Small RNA Kit (Takara, China) according to the manufacturer's protocol. Subsequently, the first-strand cDNA was synthesized for mature miRNA expression analysis by a Mir-X™ miRNA First-Strand Synthesis Kit (Code No. 638315, Takara, China). The qPCR was carried out using a miRNA SYBR Green RT-qPCR Kit (Takara, China) with the provided miRNA reference gene (U6). Relative quantitative levels were calculated based on the 2^−ΔΔ*CT*^ method (44).

### Statistical Analysis

The normal distribution and the homogeneity of the variance of growth indexes were tested, followed by one-way analysis of variance and Tukey s test. Morphological analysis between two groups was assessed by two-tailed unpaired Student's *t*-test (GraphPad). For statistically significant differences, *P* < 0.05 was required. All statistical analyses were carried out using SPSS 24.0 software. The barplot was generated by Graphpad Prism 8.0.1 software.

## Results

### Growth Performance

The survival rate (SR) was not affected by dietary treatment levels (*P* > 0.05, Table 2). The weight gain rate (WGR) of the SBM50 group was significantly lower than that of other groups (*P* < 0.05). The feed conversion ratio (FCR) of the SBM50 group was significantly higher than that of the other groups (*P* < 0.05).

**Table 2:** Growth indexes of hybrid grouper juveniles fed with different diets for 10 weeks.

**Item**	**Diet**				**PSE**	***P*-value**
	**FM**	**SBM10**	**SBM30**	**SBM50**		
WGR^1^ (%)	462.46^b^	494.28^b^	484.19^b^	383.76^a^	8.97	0.009
FCR^2^	0.96^a^	0.97^a^	0.96^a^	1.14^b^	0.01	<0.001
SR^3^ (%)	100.00	96.67	95.56	98.89	0.62	0.069

*WGR, weight gain rate; FCR, feed conversion ratio; SR, survival rate; PSE, pooled standard error*.

a, b *Mean values in the same row with different superscripts represent significant differences (P < 0.05)*.

1 *WGR (%) = 100 × (final body weight–initial body weight)/initial body weight*.

2 *FCR = total diet intake/total wet weight gain*.

3 *SR (%) = 100 × final number of fish / initial number of fish*.

### Histological Examinations and Intestinal Morphometry

**Figure 1:**
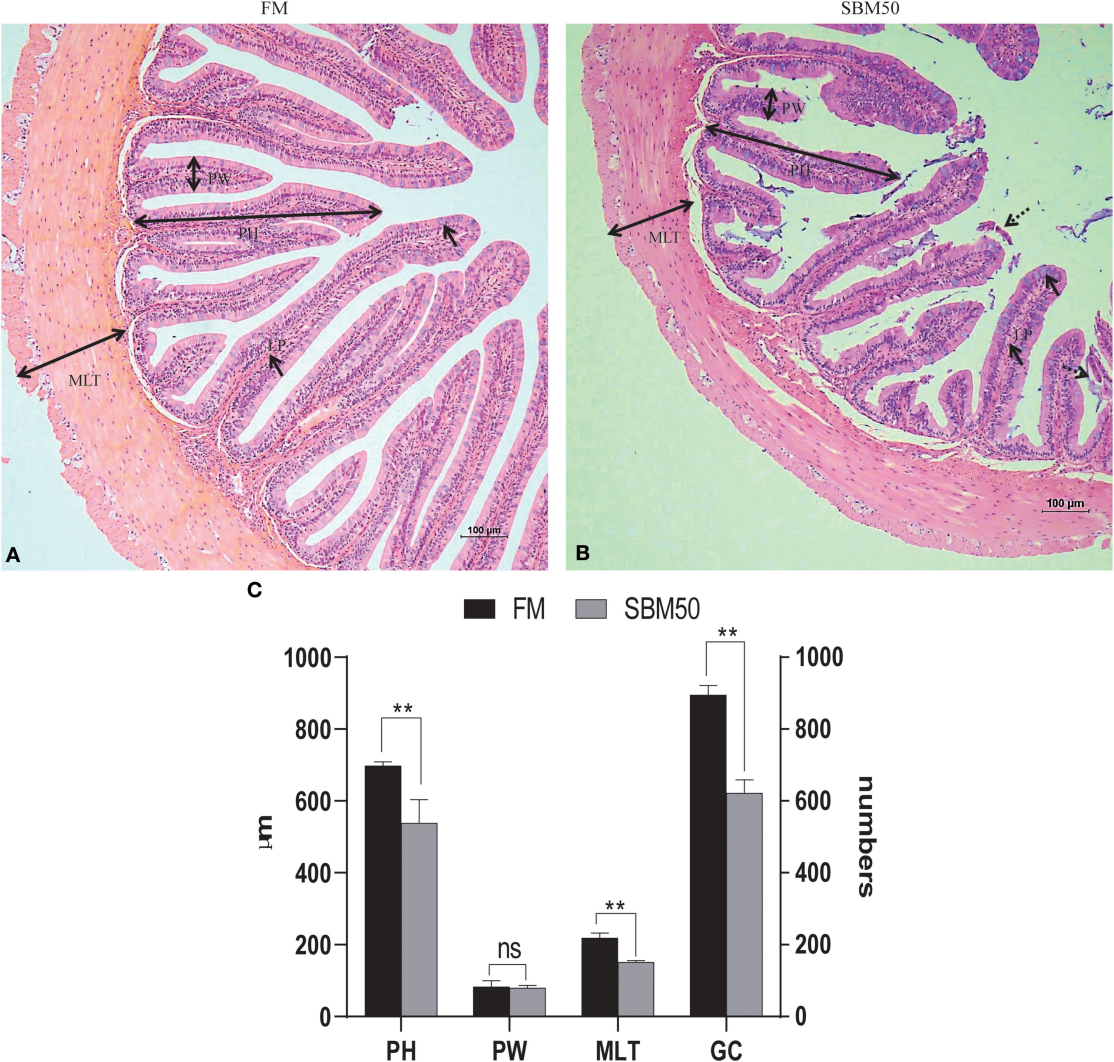
Histological analysis of the distal intestine in hybrid grouper. **(A)** HE staining of the FM group (100×). **(B)** HE staining of the SBM50 group (100×). **(C)** Intestinal morphometric measurements between FM and SBM50 groups. Mean ± S.E.M (*n* =10). LP, lamina propria; PH, Plica height; PW, plica width; MLT, muscular layer thickness; GC, goblet cells. **0.001<*P*<0.01 between two groups, NS, no significance.

More swelling of the lamina propria (LP) of the SBM50 group was observed compared with the FM group, and the intestinal villi of the SBM50 group showed signs of shedding (Figures 1A,B). The plica height (PH), muscular layer thickness (MLT), and number of goblet cells (GC) of the SBM50 group were much lower than those of the FM group (Figure 1C, *P* < 0.01). There was no significant difference in plica width (PW) between the FM and SBM50 groups (*P* > 0.05).

### Analysis of mRNA Sequencing

A total of six qualified libraries from the FM and SBM50 groups, with three biological replicates per treatment, were sequenced. An overview of the sequencing and assembly data is presented in Table 3. Approximately 33.49 and 33.6 Gb of clean reads were obtained in the FM and SBM50 groups. More than 87.1% of the reads had Q-scores at the Q30 level, and more than 75.8% of the clean reads were aligned. The length distribution of the Unigene in all six libraries is shown in Figure S1.

**Table 3:** Overview of mRNA sequencing datasets.

**Data type**	**FM_1**	**FM_2**	**FM_3**	**SBM50_1**	**SBM50_2**	**SBM50_3**
Total raw reads (M)	82.38	82.38	86.08	86.08	80.81	82.38
Total clean reads (M)	74.75	73.84	74.64	76.5	72.38	75.14
Total clean bases (Gb)	11.21	11.08	11.2	11.47	10.86	11.27
Clean reads Q30 (%)	87.81	88.19	87.92	87.18	87.5	87.69
Clean reads Q20 (%)	96.50	96.62	96.53	96.25	96.39	96.48
Total clean reads (M)	74.75	73.84	74.64	76.5	72.38	75.14
Total mapping (%)	78.9	77.55	75.76	78.06	78.22	79.02
Uniquely mapping (%)	30.94	33.08	33.26	32.34	33.72	32.05

### Differentially Expressed Genes and KEGG Pathway Analysis

**Figure 2:**
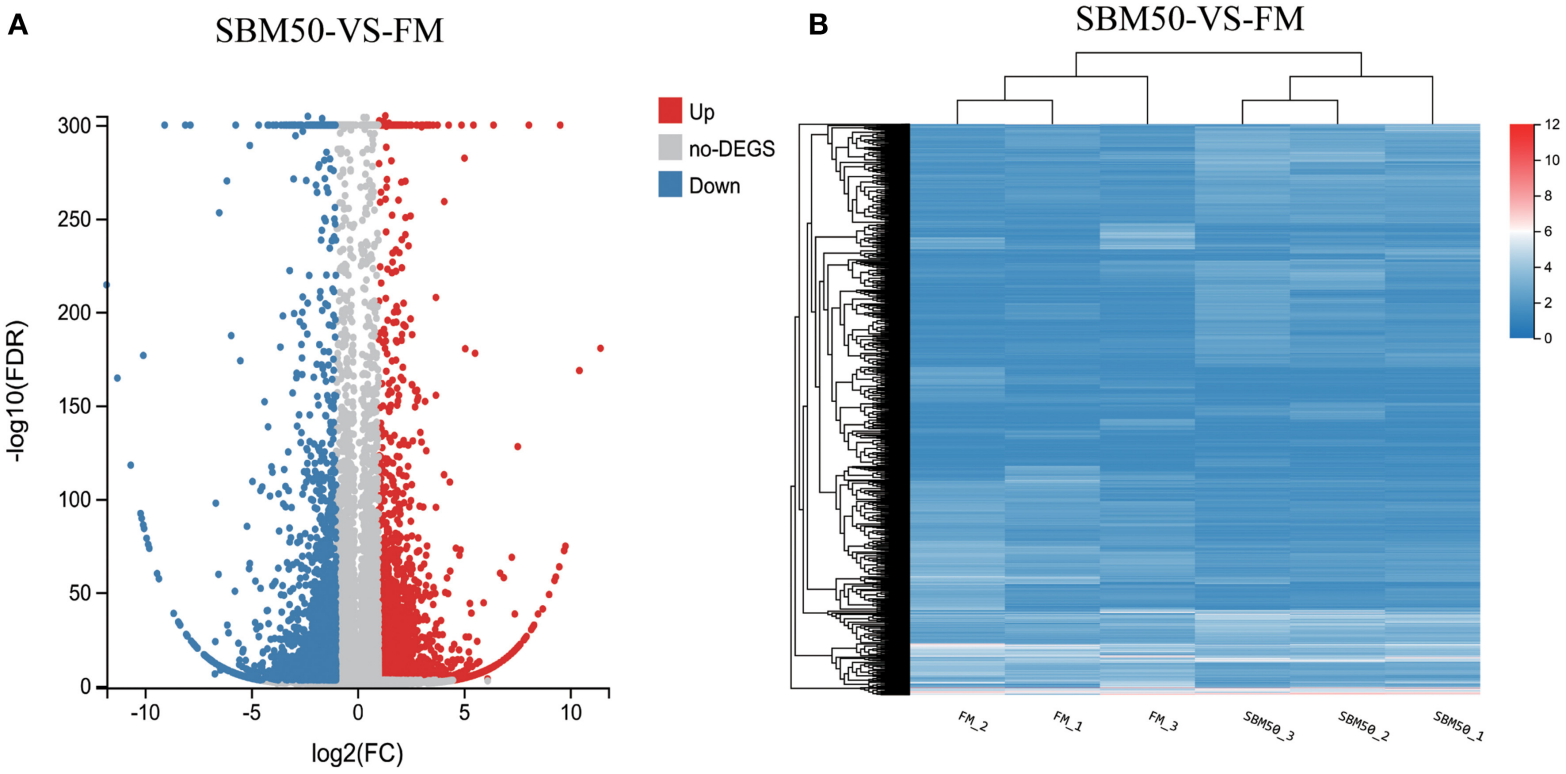
Volcano plot **(A)** and hierarchical cluster **(B)** analysis of differentially expressed genes (DEGs) in the FM and SBM50 groups. **(A)** Each point in the figure indicates a gene, the horizontal axis indicates the numerical value of DEGs, and the vertical axis indicates the negative logarithm of *P*-value-FDR. Red and blue dots represent the upregulation and downregulation of DEGs, respectively. Gray dots represent no differential expression of genes. **(B)** The color indicates the level of gene expression. Red and blue colors represent the high and low expression of genes, respectively.

**Figure 3:**
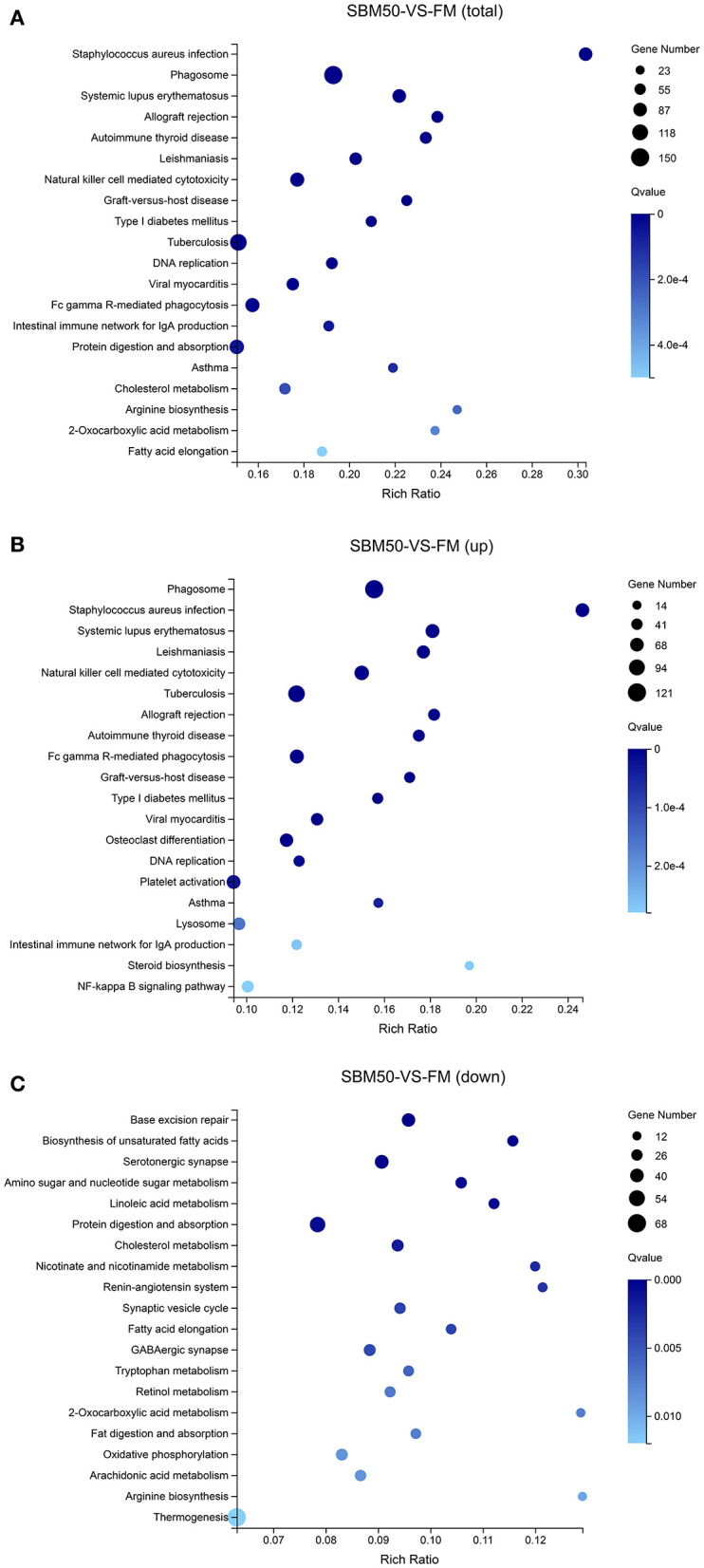
Enrichment analysis of the top 20 KEGG pathways for differentially expressed genes (DEGs). The horizontal axis represents the rich ratio, and the vertical axis represents the name of the KEGG pathway. The size of the bubble represents the number of genes annotated to the pathway. The color expresses the enriched *Q*-value. **(A)** KEGG pathways enriched by the total DEGs. **(B)** KEGG pathways enriched by the upregulated DEGs. **(C)** KEGG pathways enriched by the downregulated DEGs.

In brief, 6,390 DEGs were identified in the FM and SBM50 groups, with 3,330 upregulated and 3,060 downregulated genes (Figure 2A). The results of clustered DEGs are shown in Figure 2B. Also, 184 and 146 genes were specifically expressed in the FM and SBM50 groups, respectively (Table S2). These total DEGs were enriched in 336 pathways, and each enriched pathway contained numbers of DEGs ranging from 1 to 150 (Figure 3A). From these, the top 20 KEGG pathways were mainly involved in disease process-related pathways, such as staphylococcus aureus infection (ko05150), systemic lupus erythematosus (ko05322), allograft rejection (ko05330), autoimmune thyroid disease (ko05320), leishmaniasis (ko05140), graft-vs.-host disease (ko05332), type I diabetes mellitus (ko04940), tuberculosis (ko05152), viral myocarditis (ko05416), and asthma (ko05310), and immune system-related pathways, such as natural killer cell-mediated cytotoxicity (ko04650), Fc gamma R-mediated phagocytosis (ko04666), and the intestinal immune network for IgA production (ko04672), were significantly enriched (*Q* < 0.0001). Also, upregulated genes were significantly enriched in immune and inflammatory-related pathways such as phagosome (ko04145), natural killer cell-mediated cytotoxicity, Fc gamma R-mediated phagocytosis, the intestinal immune network for IgA production, and NF-kappa B signaling pathways (ko04064) (*Q* < 0.001, Figure 3B). The downregulated genes were mainly involved in lipid metabolisms such as fat digestion and absorption (ko04975), biosynthesis of unsaturated fatty acids (ko01040), cholesterol metabolism (ko04979), fatty acid elongation (ko00062), and linoleic acid metabolism (ko00591) (*Q* < 0.01, Figure 3C).

### Analysis of Small RNA Sequencing

Six small RNA libraries were constructed, with three biological replicates per treatment. A total of 85,789,507 and 87,445,155 raw reads were found in the FM and SBM50 groups, respectively (Table 4). Also, 77,115,163 and 81,360,125 clean reads were found in the FM and SBM50 groups, respectively. The total mapped tags to the reference genome in the FM and SBM50 groups were 71,413,180 (92.60%) and 76,824,617 (94.43%), respectively. Most small RNAs were 21–23 nt in length in all six libraries, with 22 nt being the most frequent length (Figure S2), and more than 61.7% were miRNA in the catalog of small RNA in all six libraries (Figure S3). A total of 682 mature miRNAs (Table S3) and 29 novel miRNAs (Table S4) were identified in these six small RNA libraries.

**Table 4:** Overview of small RNA sequencing datasets.

**Data type**	**FM_1**	**FM_2**	**FM_3**	**SBM50_1**	**SBM50_2**	**SBM50_3**
Raw tag count	28,825,429	28,284,361	28,679,717	29,425,359	29,689,225	28,330,571
Clean tag count	25,876,960	25,260,745	25,977,458	28,060,748	28,196,666	25,102,711
Percentage of clean tag (%)	89.77	89.31	90.58	95.36	94.97	88.61
Clean tag Q20 (%)	99.4	99.4	99.5	99.4	99.2	99
Mapped tag	24,156,912	23,426,707	23,829,561	26,376,334	26,767,611	23,680,672
Percentage of mapped tag (%)	93.35	92.74	91.73	94	94.93	94

### Differential Expression of miRNAs and Potential Target Gene Enrichment

**Figure 4:**
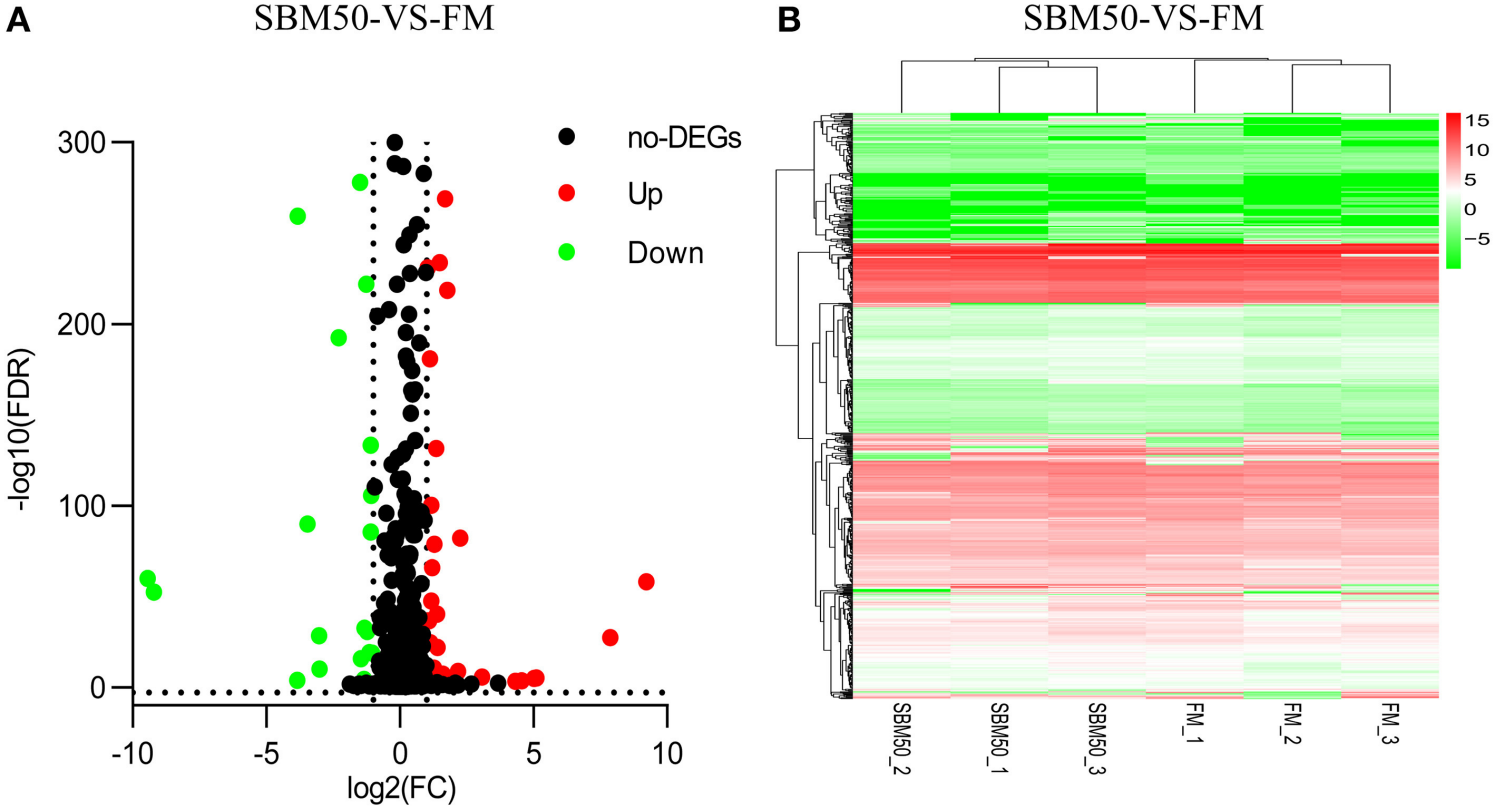
Volcano plot **(A)** and hierarchical cluster **(B)** analysis of differentially expressed miRNAs (DEmiRNAs) in the FM and SBM50 groups. **(A)** Each point in the figure indicates a gene, the horizontal axis represents the numerical value of gene expression, and the vertical axis represents the negative logarithm of *P*-value-FDR. Red and green dots indicate the upregulation and downregulation of DEmiRNAs, respectively. Black dots indicate no differential expression of genes. **(B)** The color indicates the level of gene expression. Red and green colors represent the high and low expression of genes, respectively.

Concerning the miRNA sequencing data analysis, a total of 92 DEmiRNAs were identified in the FM and SBM50 groups, with 88 known and 4 novel miRNAs (Figure 4A, Table S5). Also, 55 and 37 miRNAs were upregulated and downregulated, respectively. Cluster analysis of the DEmiRNAs was shown in Figure 4B. It was further observed that 2 miRNAs (miR-143_1, miR-21-5p) and 4 miRNAs (miR-21-5p, miR-143_1, miR-143_2, and miR-194-5p) exhibited higher expression (TPM >10,000) in the FM and SBM50 groups, respectively. Furthermore, 5 miRNAs (miR-9-3p, miR-23a-3-5p, miR-9-4-5p, novel_mir29, miR-219-5p_1, and novel_mir21) were uniquely expressed in the SBM50 group (Table S6).

**Figure 5:**
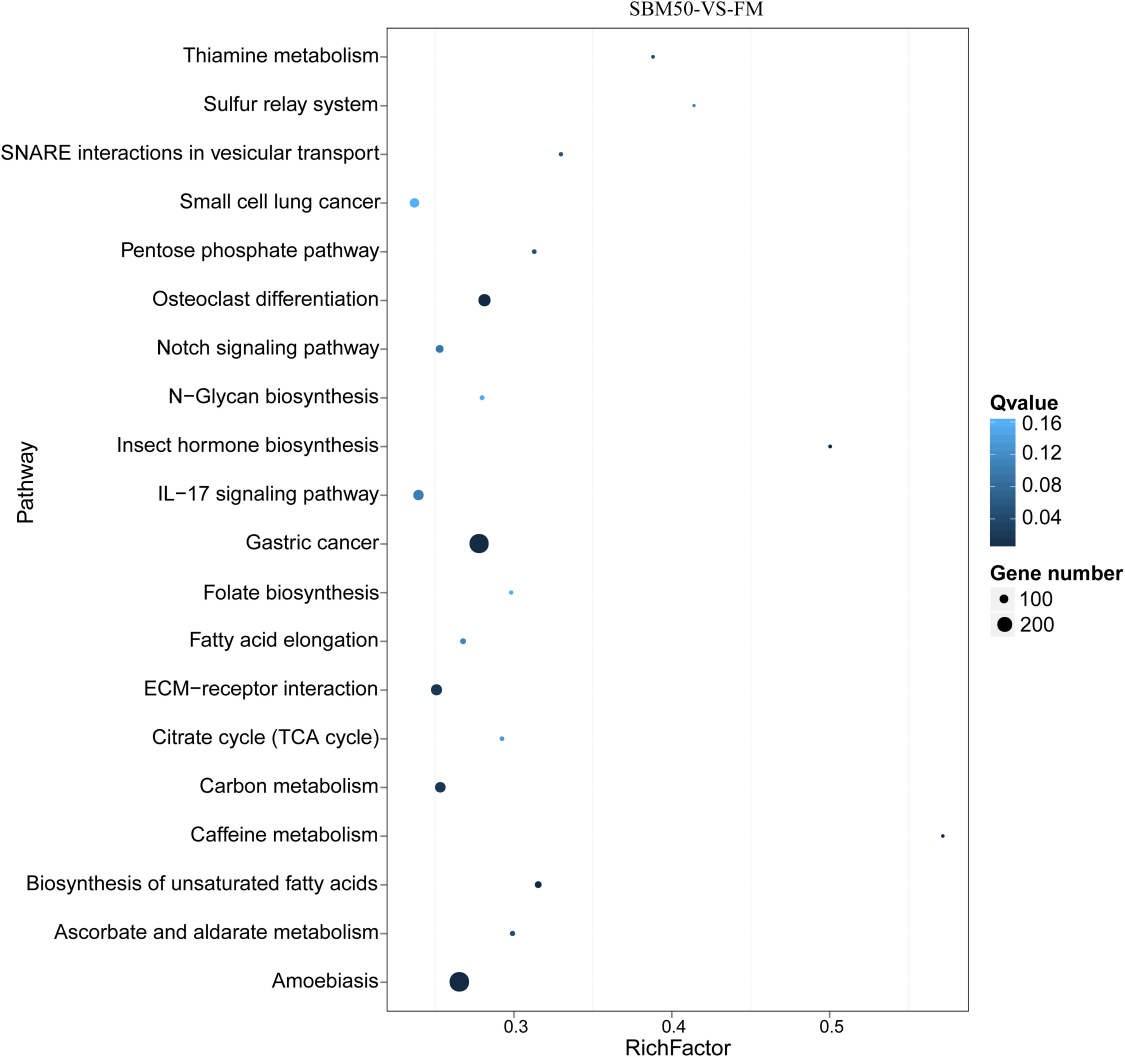
The top 20 KEGG pathways of predicted target genes in FM and SBM50 groups. The horizontal axis represents the rich ratio, and the vertical axis represents the name of the KEGG pathway. The size of the bubble represents the number of genes annotated to the pathway. The color represents the enriched *Q*-value.

In total, 15,317 target genes were predicted using the miRanda and RNAhybrid tools. The potential target genes were annotated to 340 pathways (Figure 5). The top 20 enriched pathways were most enriched in gastric cancer (ko05226), amoebiasis (ko05146), osteoclast differentiation (ko04380), ECM–receptor interaction (ko04512), and carbon metabolism (ko01200).

### Integration Analysis of the DEmiRNAs and DEGs

**Figure 6:**
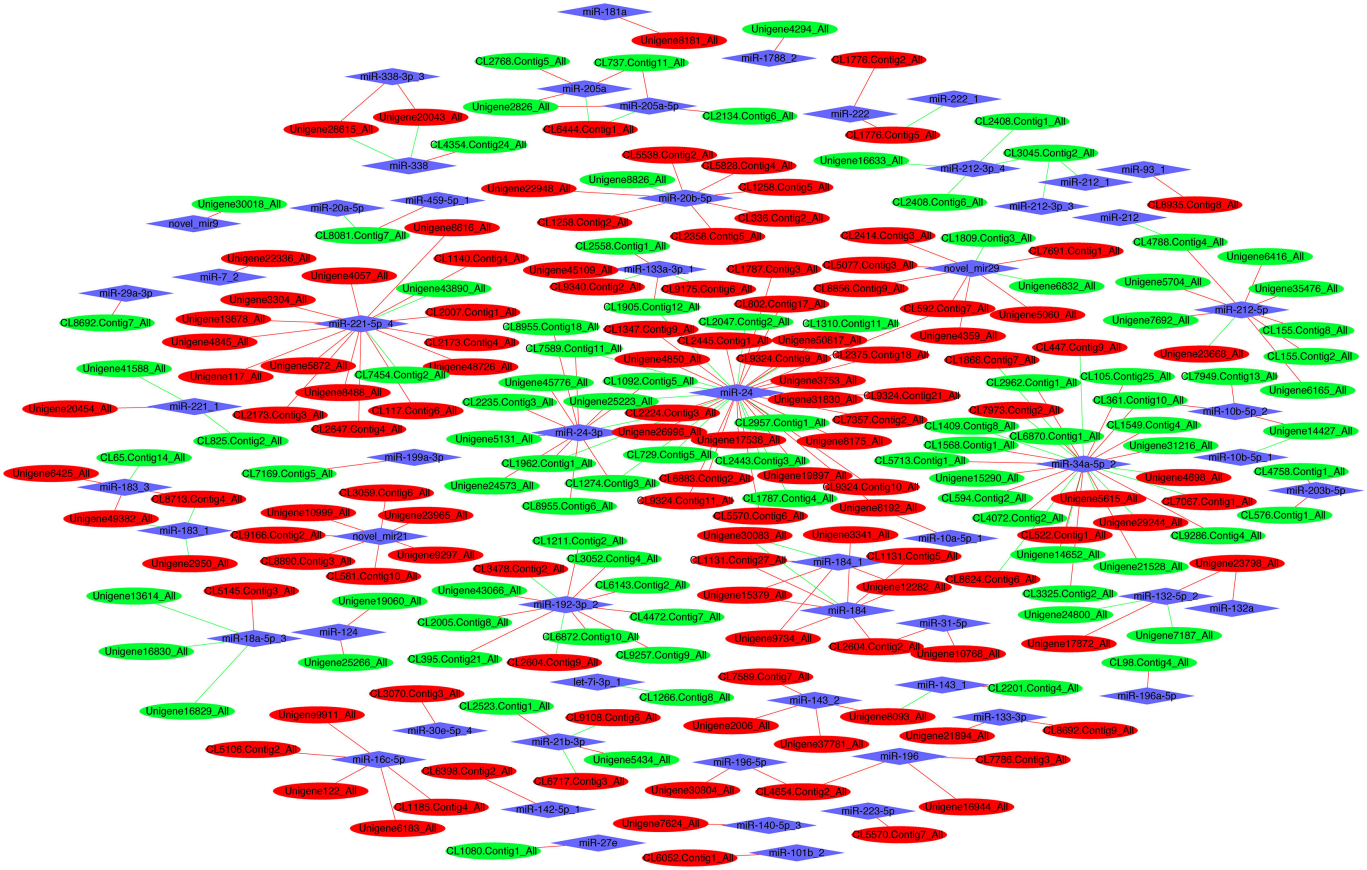
The interaction network of differentially expressed miRNAs (DEmiRNAs) combined with differentially expressed mRNAs (DEGs). Red and green ellipses represent upregulated and downregulated mRNA, respectively. Blue diamonds represent DEmiRNAs. Red and green lines represent a positive and negative correlation, respectively, between DEmiRNAs and DEGs.

A total of 244 miRNA–mRNA interactions were identified in the FM and SBM50 groups, with the involvement of 92 DEmiRNAs and 211 DEGs (Figure 6, Table S7). A positively correlated expression pattern was seen for 180 mRNA–miRNA pairs. Most miRNAs had multiple possible target genes, while different miRNAs could regulate the same target. For instance, miR-196 was the regulator of CL4654.Contig2_All, CL7786.Contig3_All, and Unigene16944_All, whereas miR-20a-5p and miR-459-5p_1 could regulate the expression of CL8081.Contig7_All.

### KEGG Enrichment Analysis of Differential Target Genes

**Figure 7:**
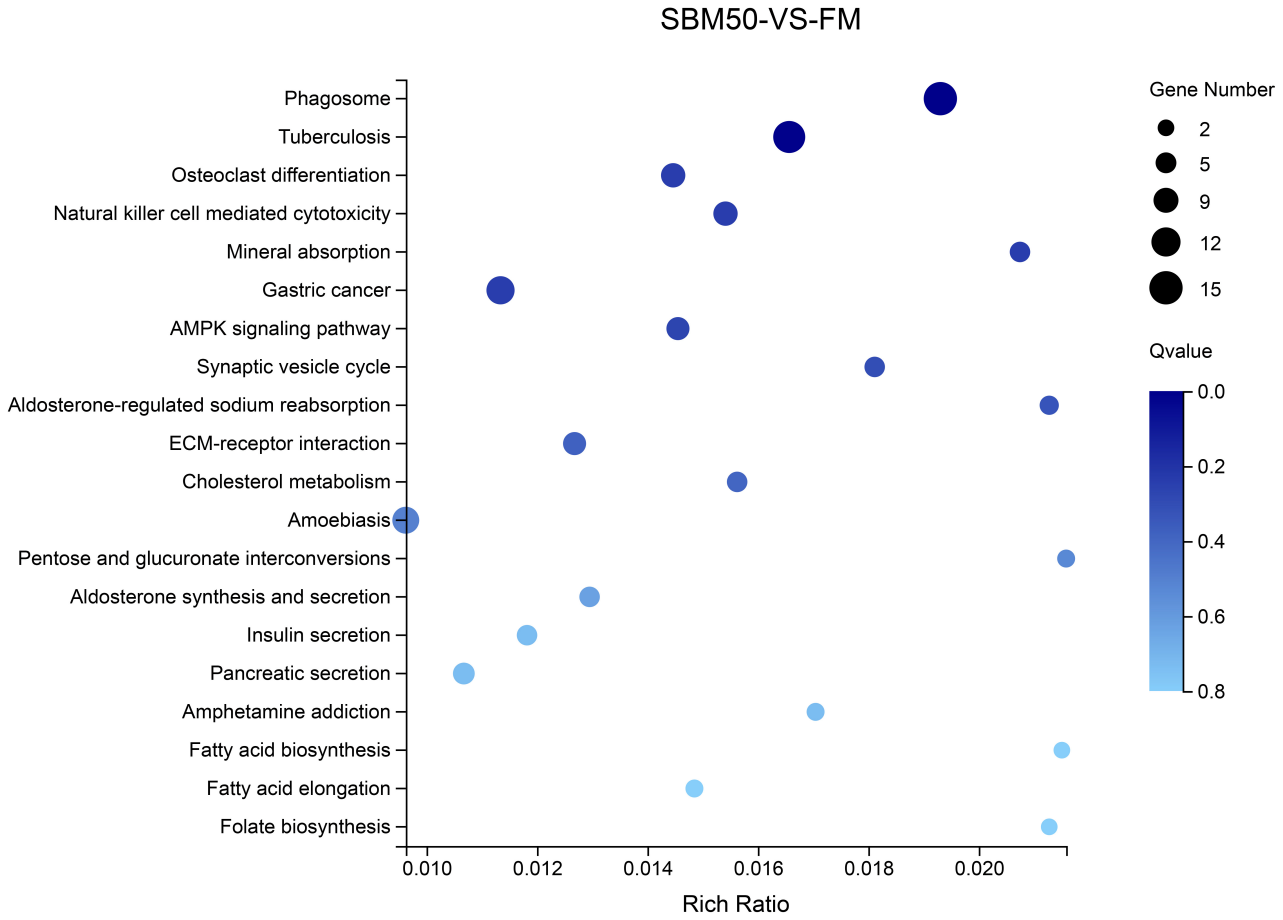
The top 20 KEGG pathways of differential target genes of differentially expressed miRNAs (DEmiRNAs) in the FM and SBM50 groups. The horizontal axis represents the rich ratio, and the vertical axis represents the name of the KEGG pathway. The size of the bubble represents the number of genes annotated to the pathway. The color indicates the enriched *Q*-value.

Differential target genes were predicted and annotated to 241 pathways. The top 5 of the top 20 KEGG pathways were phagosome, tuberculosis, osteoclast differentiation, natural killer cell-mediated cytotoxicity, and mineral absorption (ko04978), respectively (Figure 7, Table S8).

To further explain the possible immune response for SBMIE, combined with the KEGG enrichment pathway of DEGs found that 15 miRNA–mRNA pairs involved in the phagosome, 8 miRNA–mRNA pairs involved in natural killer cell-mediated cytotoxicity, 4 miRNA–mRNA pairs involved in Fc gamma R-mediated phagocytosis, and 1 miRNA–mRNA pair involved in the intestinal immune network for IgA. Details concerning the miRNA–mRNA pairs in immune-related pathways are provided in Table 5.

**Table 5:** The miRNAs and their targets involved in the immune-related pathways.

**miR-ID**	**log2FC**	**Target ID**	**log2FC**	**Pathway name**	**Blast Nr**
miR-221-5p	1.06	Unigene5872_All	1.94	Phagosome	Macrophage mannose receptor 1-like [*Fundulus heteroclitus*]
		Unigene3304_All	1.62	Phagosome	Macrophage mannose receptor 1 [*Oreochromis niloticus*]
		Unigene8616_All	1.55	Phagosome	Macrophage mannose receptor 1-like [*Lates calcarifer*]
		Unigene4845_All	1.27	Phagosome	Macrophage mannose receptor 1-like [*Lates calcarifer*]
miR-24	1.59	CL9324.Contig21_All	11.43	Phagosome	Secretory phospholipase A2 receptor-like [*Oreochromis niloticus*]
		CL9324.Contig11_All	3.69	Phagosome	Secretory phospholipase A2 receptor-like [*Oreochromis niloticus*]
		CL9324.Contig10_All	3.00	Phagosome	Snaclec stejaggregin-B subunit beta-1-like [*Lates calcarifer*]
		CL9324.Contig9_All	2.78	Phagosome	Macrophage mannose receptor 1-like [*Oreochromis niloticus*]
		CL2047.Contig2_All	−1.17	Phagosome	Galactose-specific lectin nattectin-like [*Salmo salar*]
		CL7357.Contig2_All	1.28	Phagosome	Epsilon receptor subunit alpha [*Notothenia coriiceps*]
				Fc gamma R-mediated phagocytosis	
		CL802.Contig17_All	1.78	Natural killer cell-mediated cytotoxicity	Lymphocyte cytosolic protein 2-like [*Larimichthys crocea*]
		Unigene31830_All	2.25	Natural killer cell-mediated cytotoxicity	Perforin-1-like [*Lates calcarifer*]
miR-18a-5p_3	1.18	Unigene16830_All	−1.41	Phagosome	Platelet glycoprotein 4 [*Lates calcarifer*]
		Unigene16829_All	−1.17	Phagosome	Platelet glycoprotein 4 [*Lates calcarifer*]
		CL5145.Contig3_All	2.10	Phagosome	Gamma Fc region receptor II-a-like [*Lates calcarifer*]
				Natural killer cell-mediated cytotoxicity	
				Fc gamma R-mediated phagocytosis	
miR-199a-3p	−9.93	CL7169.Contig5_All	−1.76	Natural killer cell-mediated cytotoxicity	Connective tissue growth factor-like [*Paralichthys olivaceus*]
				Fc gamma R-mediated phagocytosis	
novel_mir29	5.03	Unigene4359_All	1.30	Phagosome	Scavenger receptor class B member 1 isoform X1 [*Lates calcarifer*]
		CL2414.Contig3_All	1.03	Natural killer cell-mediated cytotoxicity	ZAP70, partial [*Epinephelus coioides*]
miR-205a-5p	−3.46	CL2134.Contig6_All	−3.46	Phagosome	Gamma Fc region receptor II-like isoform X1 [*Lates calcarifer*]
miR-133-3p	2.61	Unigene21894_All	1.19	Natural killer cell-mediated cytotoxicity	SH2 domain-containing protein 3C-like [*Stegastes partitus*]
miR-16c-5p	1.87	Unigene6183_All	1.14	Natural killer cell-mediated cytotoxicity	Interferon alpha/beta receptor 1-like, partial [*Notothenia coriiceps*]
miR-192-3p	−1.11	CL3478.Contig2_All	1.00	Natural killer cell-mediated cytotoxicity	SH3 domain-binding protein 2 isoform X3 [*Lates calcarifer*]
miR-205a-5p	−3.46	CL2134.Contig6_All	−3.46	Fc gamma R-mediated phagocytosis	Gamma Fc region receptor II-like isoform X1 [*Lates calcarifer*]
miR-20b-5p	1.83	Unigene8826_All	−1.30	Intestinal immune network for IgA production	Hepatitis A virus cellular receptor 2 homolog [*Neolamprologus brichardi*]

### Real-Time Quantitative PCR Validation

**Figure 8:**
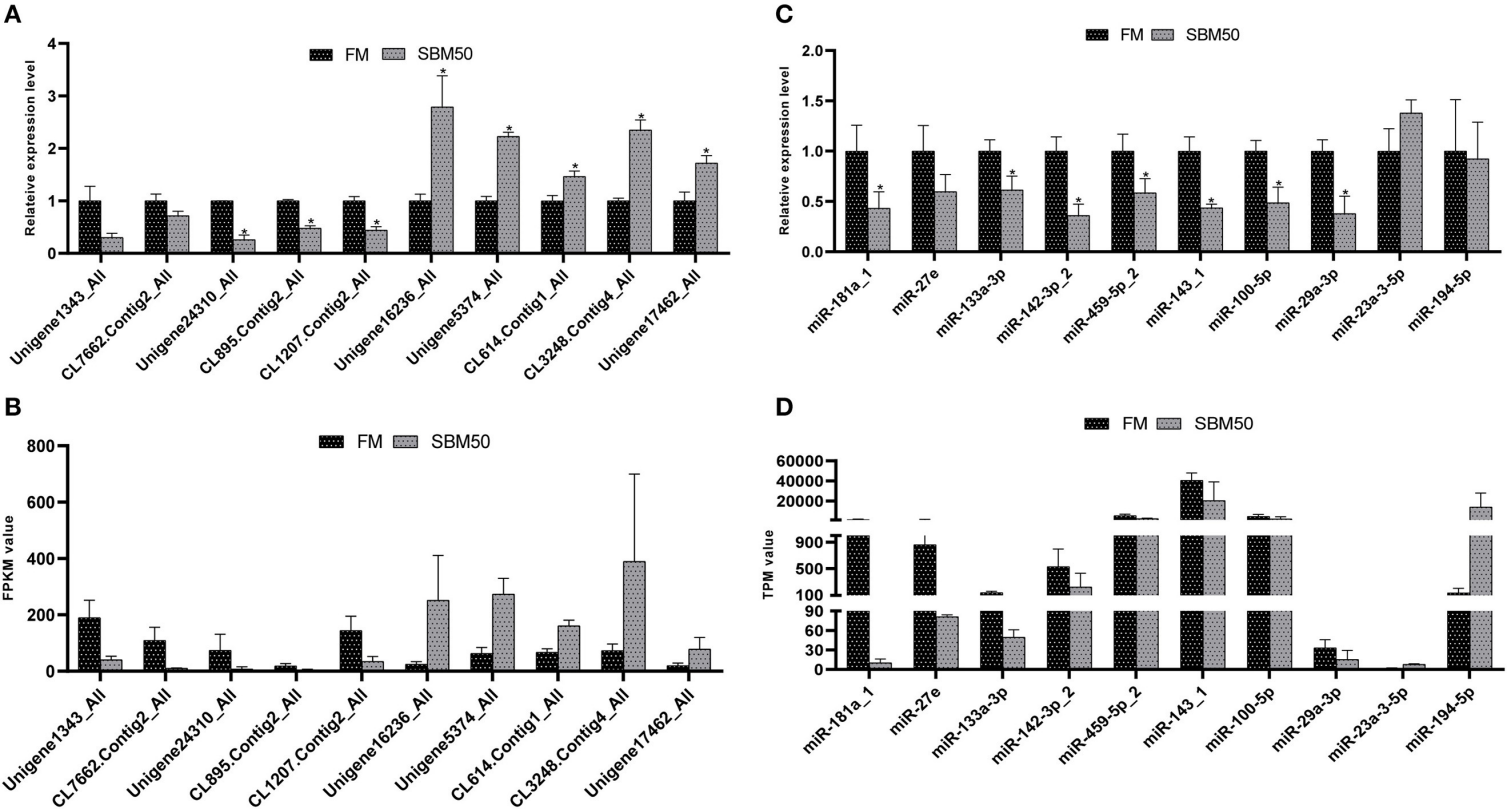
RT-qPCR validation of differentially expressed miRNAs (DEmiRNAs), and genes (DEGs) in the FM and SBM50 groups. **(A)** The relative expression levels of mRNA between the two groups. **(B)** The value of fragments per kilobase of transcript sequence per million base pairs sequenced (FPKM) of the DEGs between the two groups. **(C)** The relative expression levels of the DEmiRNAs between the two groups. **(D)** The transcripts per million (TPM) values of miRNA between the two groups. The results of **(A,C)** are presented as the mean ± S.E.M (*n* =3). The sequencing results of **(B,D)** are presented as the mean. *P < 0.05 between two groups.

In total, 10 miRNAs and 10 mRNAs were selected to test their expression and the results suggested that DEmiRNAs except for miR-194-5p and DEGs showed a similar expression pattern to the high-throughput sequencing data (Figure 8). There were no significant differences in expression levels of mRNA genes (Unigene1343_All and CL7662.Contig2_All) and miRNA (miR-27e, −23a-3-5p, and miR-194-5p) between the two groups (*P* > 0.05). Also, fold-changes between miRNA or mRNA and their respective qPCR results correlated well (Table S9).

## Discussion

The nutritional trial of this study revealed that a 22.4% SBM, i.e., 30% substitution of FM protein, did not significantly influence the growth of hybrid grouper compared to the FM diet. However, the effect of SBM on growth was substitution-related, and the growth performance became progressively compromised with increasing SBM substitution level, which is consistent with the previous studies (45, 46). In this study, growth performance was significantly reduced when dietary SBM content reached 370 g/kg, i.e., 50% substitution of the FM protein. Hybrid grouper fed with SBM replacing 50% of the FM protein diet showed swelling of the lamina propria and reduction of plica height, muscular layer thickness, and number of goblet cells in the distal intestine of hybrid grouper, indicating that enteritis and intestinal injury had appeared. The severity of histopathological changes observed under SBM application relies on the level of soybean inclusion. SBMIE is now commonly used as a model for the study of intestinal inflammation in fish (6, 7, 47). The histopathological changes of SBMIE have been widely researched and are characterized by a swelling of the subepithelial mucosa and lamina propria, a reduced mucosal fold height, a profound infiltration of various inflammatory cells, and loss of normal enterocyte supranuclear absorptive vacuolization (13, 31, 48). Liu et al. (10) found that turbot fed with SBM replacing 40% of the fish meal protein diet showed obvious enteropathy, including a reduction of absorptive surface and obvious infiltration of mixed leukocytes in the lamina propria.

To reveal the underlying immune response of fish SBMIE, 6,390 DEGs in the distal intestine of hybrid grouper were identified in this study. Also, the enhanced gene expressions were found to involved in immune- and inflammatory-related pathways, including phagosome, natural killer cell-mediated cytotoxicity, the intestinal immune network for IgA production, Fc gamma R-mediated phagocytosis, and the NF-kappa B signaling pathway. The above results suggested that these pathways played a vital role in fish SBMIE. Similar immune-related pathways have been reported in SBMIE in other carnivorous fish, such as salmon (16, 49) and turbot (6, 7). It is worth noting that the downregulated gene expression was in KEGG pathways mainly involved in lipid metabolisms such as biosynthesis of unsaturated fatty acids, linoleic acid metabolism, cholesterol metabolism, fat digestion, absorption, and arachidonic acid metabolism. This may indicate that impaired lipid metabolism could be a consequence of “tissue malfunction” (47, 49). The results enable a better understanding of why LC-PUFA biosynthesis, cholesterol biosynthesis, lipid digestion, and the PPAR signaling pathway of distal intestine were influenced when Atlantic salmon ingested feed in which the fish meal was partially replaced by soybean meal (1, 16).

Despite the major contribution of mRNAs, miRNAs also play a key role during immune processes in fish SBMIE. In this study, predicted target genes for DEmiRNAs are annotated to 340 signaling pathways. Immune-related pathways, including ECM–receptor interaction, the NF-kappa B signaling pathway, and the IL-17 signaling pathway, were enriched. The downregulation of miR-192-3p and miR-212-5p expression involved in the regulation of the ECM–receptor interaction pathway. The down-regulation of genes involved in the ECM–receptor interaction pathway in response to SBM stress was also reported in Grass carp (5). To further introduce miRNAs in detail, enteritis-related miRNAs have been found both in human and mammals. The present miRNA results include inflammatory bowel diseases (IBD) related-miRNAs, such as miR-124, miR-24, miR-221, and miR-132. The results of this study showed that upregulation of miR-124 expression and downregulation of miR-24, miR-221, and miR-132 expression were observed in the SBM50 group (Table S5). Similar expression patterns were found in colon tissues of children with active ulcerative colitis (UC), where decreased levels of miR-124 appeared to enhance expression and activity of STAT3, which could induce inflammation and pathogenesis (50). There were elevated levels of miR-24, miR-221, and miR-132 in colonic biopsies from UC, which suggested that they are an important regulator of the intestine barrier that may be essential in the pathogenesis of IBD (51). The above results indicated that miR-124, miR-24, miR-221, and miR-132 may play important roles in SBMIE of hybrid grouper juveniles, which may suggest their therapeutic potential.

To further explain the possible immune response for SBMIE through integrative transcriptomic and small RNA sequencing, the KEGG enrichment pathways of differential target genes were analyzed. Combined with the KEGG enrichment pathways of DEGs, it was concluded that immune-related signaling pathways such as phagosome, natural killer cell-mediated cytotoxicity, Fc gamma R-mediated phagocytosis, and the intestinal immune network for IgA production were enriched. The most enhanced gene expression being in the phagosome pathway suggested the involvement of a macrophage as the main intestine phagocyte during enteritis (52). A total of 14 miRNA–mRNA pairs also suggested that the phagosome pathway could play a key role in intestinal inflammation. In addition, this study identified 92 DEGs related to the natural killer cell-cytotoxicity pathway. The role of this pathway in the immune response to pathogens has also been reported in different fish species such as large yellow croaker (*Larimichthys crocea*) (53) and half-smooth tongue sole (*Cynoglossus semilaevis*) (54). A previous study reported that mammalian natural killer (NK) cells mediated cytotoxic activity via two distinct pathways (55). NK cells can release cytotoxic granules, including perforin and granzymes, on the surface of diseased cells. Granzymes can then stimulate caspase activation, mitochondrial dysfunction, or apoptosis (55). In this study, the upregulation of *granzyme* (gene id: Unigene24888_All) and *perforin* (gene id: Unigene31830_All, Blast Nr annotated in Table 5) gene expressions indicated that granule-mediated cytotoxicity may be triggered by the targeted release of lytic granules toward a locally attached target cell. Wu et al. (5) reported that the intestinal immune network for the IgA production pathway was upregulated in the early stages of Grass carp in response to high-SBM-content stress. Similar results were also reported in the present study: most genes in pathways of Fc gamma receptor-mediated phagocytosis and the intestinal immune network for IgA production were significantly upregulated. The role of the two pathways in immune response to pathogens has also been reported in fish such as half-smooth tongue sole (53), large yellow croaker (54), and darkbarbel catfish (Pelteobagrus vachellii) (56). Previous studies reported that the target genes of the DEmiRNAs were involved in pathways of Fcγ R-mediated phagocytosis and the intestinal immune network for IgA production (57, 58). In this study, related miRNA–mRNA pairs were also enriched in the two pathways mentioned above (Table 5). Thus, our results suggested that phagosome, natural killer cell-mediated cytotoxicity, Fc gamma R-mediated phagocytosis, and the intestinal immune network for IgA production pathways may play a vital role in SBMIE of carnivorous fish.

In conclusion, our study is the first to offer the transcriptomic and small RNA profiles for SBMIE in hybrid grouper. Overall, 6,390 mRNAs and 92 miRNAs were differentially expressed under dietary SBM stress. Our findings support the notion that DEmiRNAs and their target mRNAs play an important role in immune regulation. Also, investigation of KEGG enrichment pathways by integrative transcriptomic and small RNA profiling revealed that the immune mechanism for SBMIE in hybrid grouper may be associated with the phagosome, natural killer cell-mediated cytotoxicity, Fc gamma R-mediated phagocytosis, and the intestinal immune network for IgA production pathways. Our findings offer important insights for the understanding of the RNA profiles and further elucidation of the underlying molecular immune mechanism for SBMIE in carnivorous fish.

## Data Availability Statement

The datasets presented in this study can be found in online repositories. The names of the repository/repositories and accession number(s) can be found below: https://www.ncbi.nlm.nih.gov/, SUB7020170; https://www.ncbi.nlm.nih.gov/, SUB7175134.

## Ethics Statement

The animal study was reviewed and approved by The Animal Research and Ethics Committees of the Guangdong Ocean University, China.

## Author Contributions

BT and SC conceived the project. GY performed animal experiments. XD bought feed ingredients. SZ bought sample analysis regent kits. HL and QY managed and repaired experimental instruments. YH did data analysis and wrote the manuscript. SC revised the manuscript. All authors contributed to the article and approved the submitted version.

## Conflict of Interest

The authors declare that the research was conducted in the absence of any commercial or financial relationships that could be construed as a potential conflict of interest.
