# Supplementary material for: Integrative Transcriptomic and Small RNA Sequencing Reveals Immune-Related miRNA–mRNA Regulation Network for Soybean Meal-Induced Enteritis in Hybrid Grouper, Epinephelus fuscoguttatus♀ × Epinephelus lanceolatus♂
Source: Front Immunol. 2020 Aug 6;11:1502. doi: 10.3389/fimmu.2020.01502 (PMC7438716; doi:10.3389/fimmu.2020.01502)
Supplement: Supplementary file 1 [file Data_Sheet_1.ZIP › Supplementary Figures and Tables/Supplementary Figure.pdf]

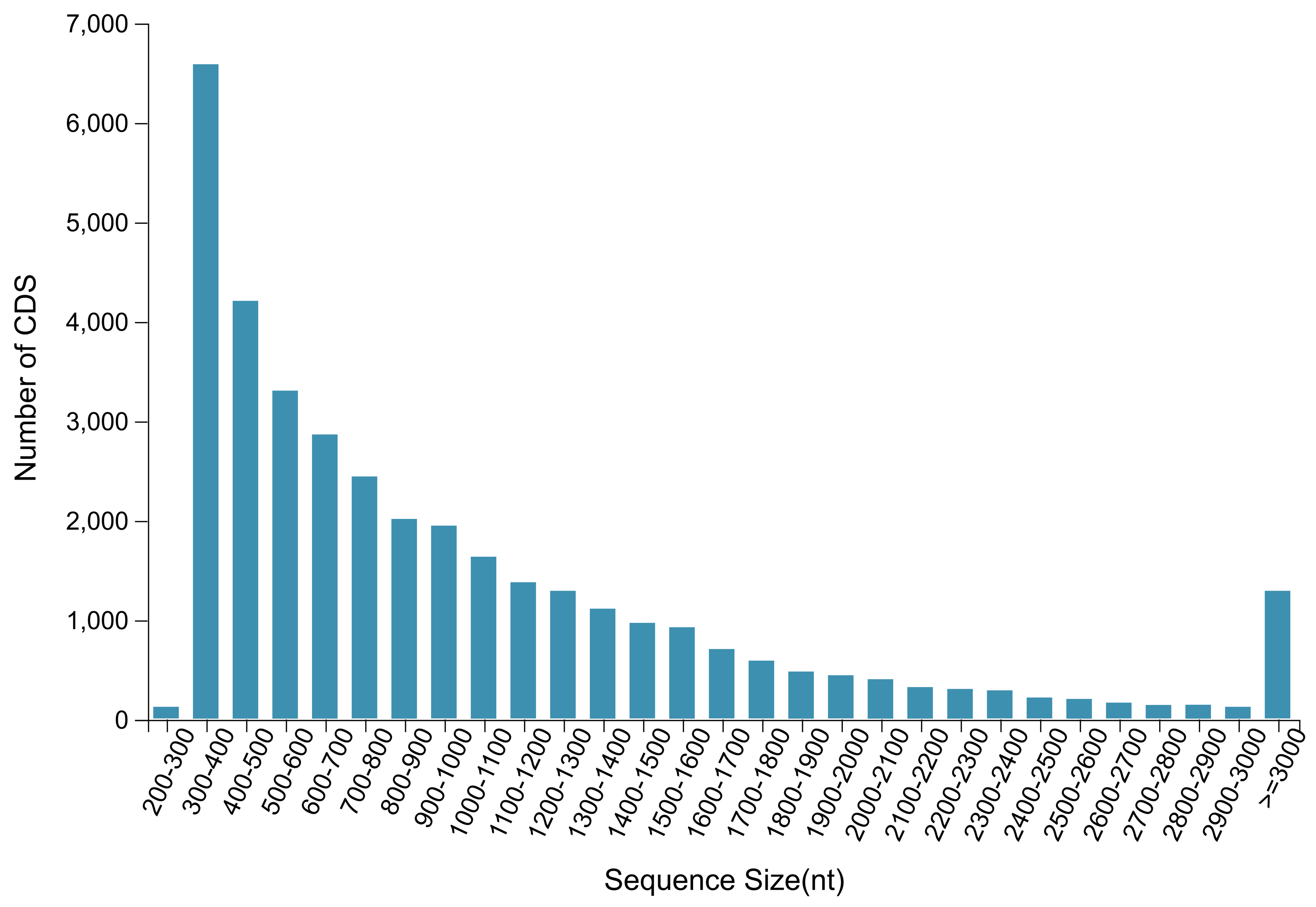

Figure S1 Length distribution of mRNA

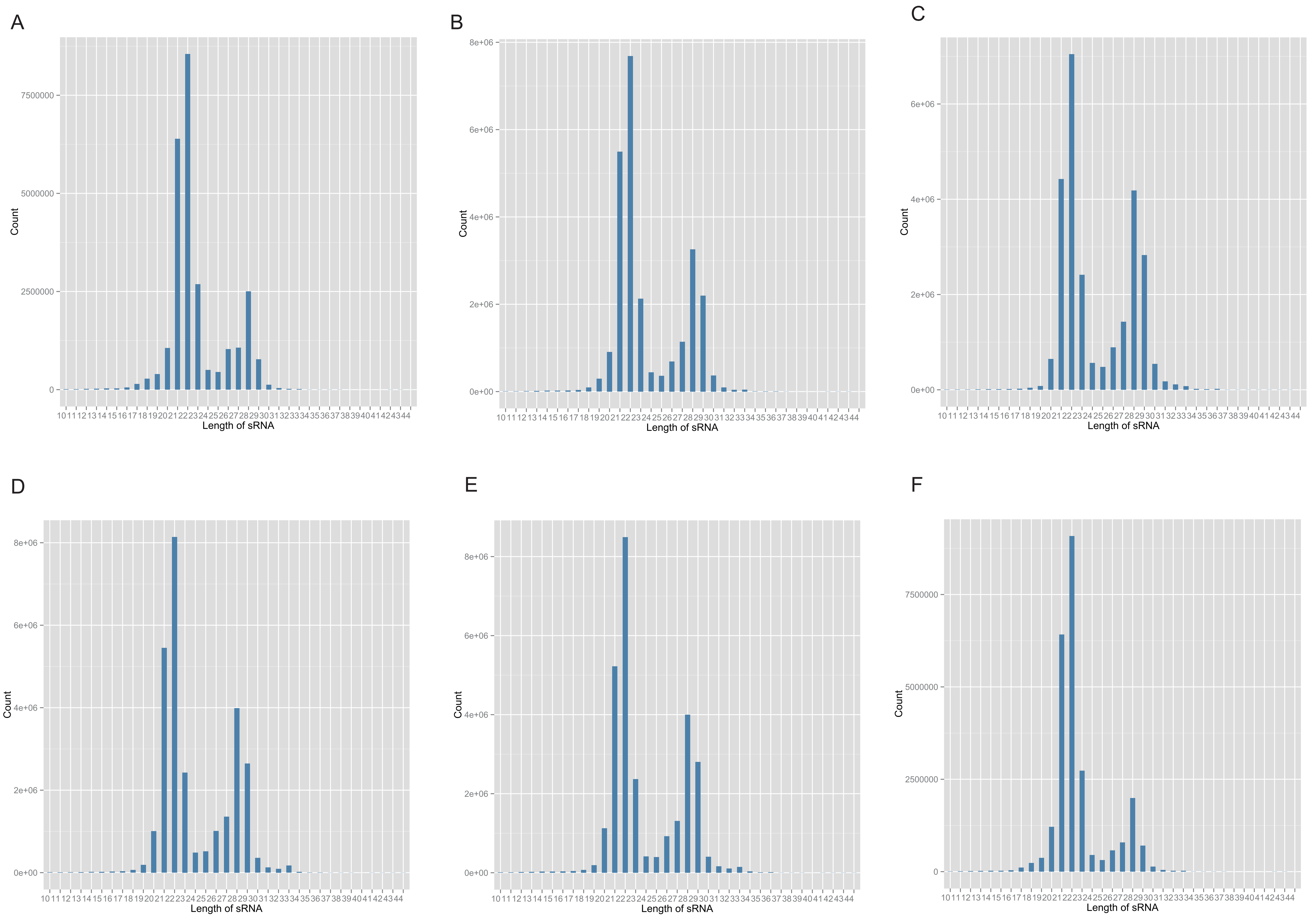

Figure S2 Size distribution of sRNA. A, FM_1; B, FM_2; C, FM_3; D, SBM50_1; E, SBM50_2; F, SBM50_3.

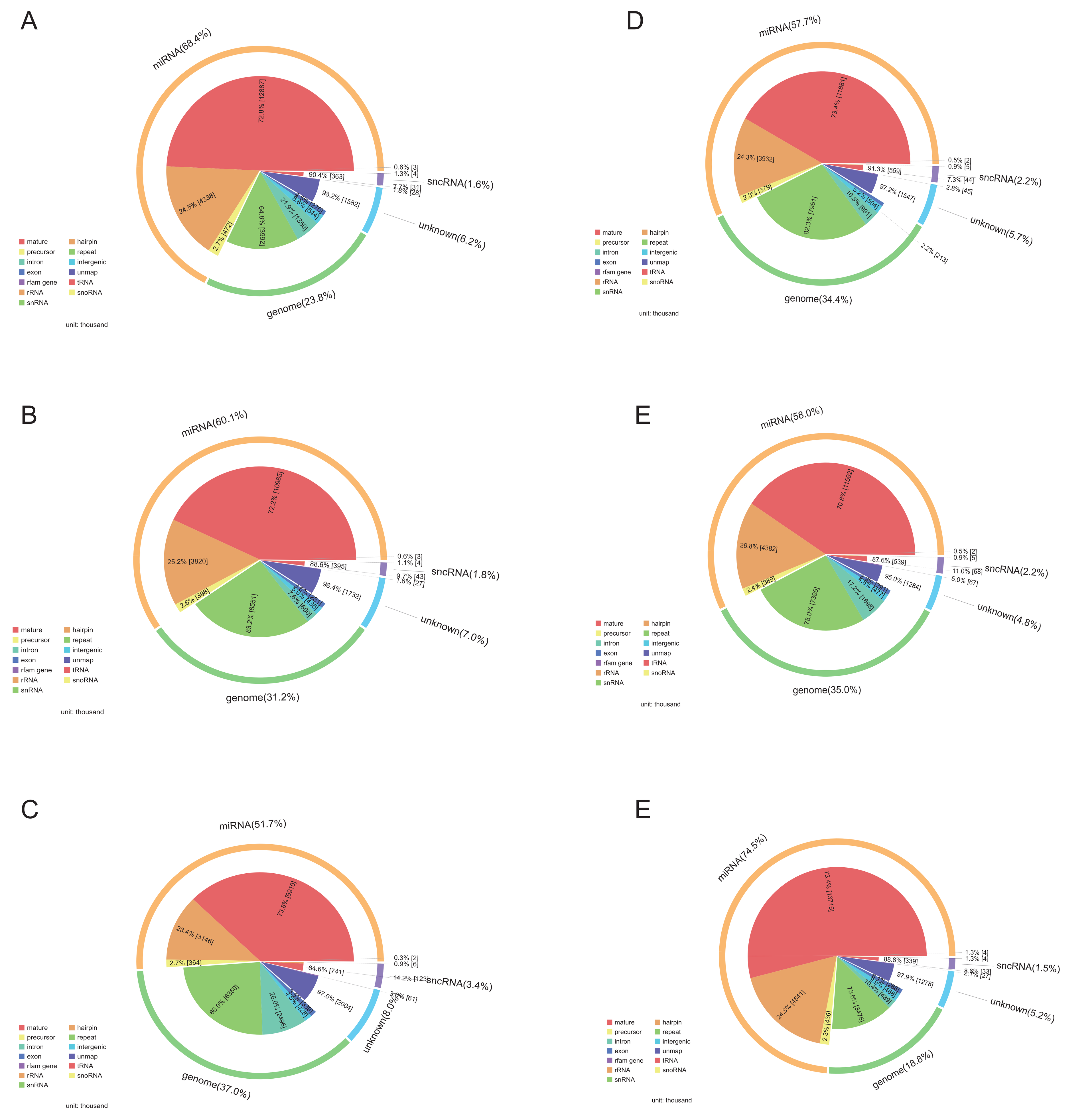

Figure S3 Catalog of sRNA. A, FM_1; B, FM_2; C, FM_3; D, SBM50_1; E, SBM50_2; F, SBM50_3.
